# Drug-induced anaphylaxis in China: a 10 year retrospective analysis of the Beijing Pharmacovigilance Database

**DOI:** 10.1007/s11096-017-0535-2

**Published:** 2017-10-31

**Authors:** Ying Zhao, Shusen Sun, Xiaotong Li, Xiang Ma, Huilin Tang, Lulu Sun, Suodi Zhai, Tiansheng Wang

**Affiliations:** 1 Department of Pharmacy, Peking University Third Hospital, Beijing, China; 2 Department of Pharmacy, Beijing Shijitan Hospital, Beijing, China; 3 Department of Pharmacy Administration and Clinical Pharmacy, School of Pharmaceutical Sciences, Peking University, Beijing, China; 4 College of Pharmacy and Health Sciences, Western New England University, Springfield, Massachusetts, USA; 5 Department of Epidemiology, Richard M. Fairbanks School of Public Health, Indiana University–Purdue University Indianapolis, Indianapolis, IN USA; 6 Department of Epidemiology, University of North Carolina at Chapel Hill, Chapel Hill, North Carolina USA

**Keywords:** China, Clinical features, Drug category, Drug-induced anaphylaxis, Pharmacovigilance

## Abstract

*Background* Few studies on the causes of drug-induced anaphylaxis (DIA) in the hospital setting are available. *Objective* We aimed to use the Beijing Pharmacovigilance Database (BPD) to identify the causes of DIA in Beijing, China. *Setting* Anaphylactic case reports from the BPD provided by the Beijing Center for Adverse Drug Reaction Monitoring. *Method* DIA cases collected by the BPD from January 2004 to December 2014 were adjudicated. Cases were analyzed for demographics, causative drugs and route of administration, and clinical signs and outcomes. *Main outcome measure* Drugs implicated in DIAs were identified and the signs and symptoms of the DIA cases were analyzed. *Results* A total of 1189 DIA cases were analyzed. The mean age was 47.6 years, and 732 (61.6%) were aged from 18 to 59 years. A total of 627 patients (52.7%) were females. There was a predominance of cardiovascular (83.8%) followed by respiratory (55.4%), central nervous (50.1%), mucocutaneous (47.4%), and gastrointestinal symptoms (31.3%). A total of 249 different drugs were involved. DIAs were mainly caused by antibiotics (39.3%), traditional Chinese medicines (TCM) (11.9%), radiocontrast agents (11.9%), and antineoplastic agents (10.3%). Cephalosporins accounted for majority (34.5%) of antibiotic-induced anaphylaxis, followed by fluoroquinolones (29.6%), beta-lactam/beta-lactamase inhibitors (15.4%) and penicillins (7.9%). Blood products and biological agents (3.1%), and plasma substitutes (2.1%) were also important contributors to DIAs. *Conclusion* A variety of drug classes were implicated in DIAs. Patients should be closely monitored for signs and symptoms of anaphylaxis when medications are administered especially with antibiotics, TCM, radiocontrast and antineoplastic agents.

**Electronic supplementary material:**

The online version of this article (doi:10.1007/s11096-017-0535-2) contains supplementary material, which is available to authorized users.

## Impacts on practice

- Drug-induced anaphylaxis accounts for at least 12% of adverse drug event reports in Chinese hospitals collected by the Beijing Pharmacovigilance Database between 2004 to 2014, and the associated mortality rate is 3.3%.
- The top four drug categories implicated in drug induced anaphylaxis cases in China are antibiotics, traditional Chinese medicines, radiocontrast media and antineoplastic agents.
- Drug induced anaphylaxis most often presents with cardiovascular system symptoms, followed by mucocutaneous, respiratory and central nervous system symptoms.

## Introduction

Anaphylaxis is a severe, life-threatening, systemic allergic reaction that occurs rapidly after contact with an inducing substance. Common triggers of anaphylaxis include food, insect stings, drugs and latex [1, 2]. Susceptibility (or incidence) of anaphylaxis varies with age, allergen exposure, and predisposing genetic factors [1, 3]. Symptoms of anaphylaxis may progress rapidly and involve multiple target organ systems including the integumentary, respiratory, gastrointestinal, and cardiovascular systems [1].

Of the most common triggers of anaphylaxis [1, 2], drugs are considered to be the primary triggers in adults [1, 4]. Administration of any drug by any route can potentially cause anaphylaxis [2, 5]. According to a retrospective U.S. epidemiology study, medications were the most common cause (58.8%) of 2458 anaphylaxis-related deaths from 1999 to 2010 [6]. Furthermore, a multicenter retrospective study from Korea, drug-induced anaphylaxis (DIA) accounted for 46.5% of all 1806 anaphylaxis cases, becoming the most common trigger of anaphylaxis in Korea [7].

Although the epidemiological data of DIA have been reported in western countries [6, 8, 9], data is limited in Asian population. Most published studies are case reports or case series focused on specific drugs such as antibiotics or special clinical situations for instance during perioperative procedures. Studies are needed to confirm the previous findings and to add new knowledge to this area in Asian population. Our previous study assessed the use of epinephrine in managing patients with DIA through the analysis of the Beijing Pharmacovigilance Database (BPD) [10], and the present study is an extension of this project to provide a detailed analysis of the reported DIA cases.

## Aim of the study

The objective of this study was to contribute to a better understanding of DIAs in Beijing, China, based on anaphylaxis case reports by the BPD over a decade period. The following information was extracted and analyzed: causative drugs, clinical features and severities of DIA cases.

## Ethics approval

This study was considered to be exempt from further review by the Institutional Review Board, Peking University Third Hospital. Patient informed consent was not required because this was a retrospective study using only de-identified data.

## Method

Using a structured database inquiry, extraction, and case adjudication methodology as reported in our previous study [10], we performed a detailed analysis on DIA cases. The cases were reported to the BPD from January 1, 2004 to December 31, 2014.

In contrast with our previous study [10], anaphylaxis-inducing drugs were classified into various pharmacotherapeutic groups according to the Martindale—The Complete Drug Reference (37th edition) [11], World Health Organization (WHO) Model Formulary (2008) [12], and Chinese Pharmacopoeia (ChP) [13]. Within each group there were several subgroups. Previously, cases in which more than one drug was suspected were defined as “Associations” [10]. In this study, we further classified drugs under the “Associations” according to the original judgments of physicians when information is available. For example, a case was reported in which two drugs were administered to a patient-Ambroxol injection and Lomefloxacin injection. Clinicians filing the report deemed that Lomefloxacin injection was more likely to induce anaphylaxis. Accordingly, we classified this case into “Antibiotics” instead of “Associations”. For each DIA case, we assessed the case severity into three grades: grade 1 category was patients with only cutaneous involvement, grade 2 included patients with mild-to-moderate manifestations of anaphylaxis, and those with grade 3 reactions had severe presentations with cutaneous, gastrointestinal, and potentially life-threatening respiratory or cardiovascular signs and symptoms [14].

### Statistical analysis

The statistical analysis was performed using the SPSS version 22 (SPSS Inc., IL, USA). Continuous variables were subjected to normality tests using the single sample Kolmogorov–Smirnov test, where data in accordance with normal distribution was expressed as mean ± standard deviation, while those in accordance with the non-normal distribution was expressed as median (min, max), and the dichotomous variables were described as frequency (percentage).

## Results

### Demographic and clinical characteristics

A total of 9425 patients with drug-induced hypersensitivity reactions were identified from the BPD. After initial screening and adjudication, 1189 patients were ultimately included in our analysis [10]. Of these patients, the mean age was 47.6 years, 732 (61.6%) were aged from 18 to 59 years. A total of 627 (52.7%) were female patients (Table 1).

**Table 1:** Demographics, clinical characteristics and outcome of patients with drug-induced anaphylaxis

Variable	Value, no. (%)	95% CI
Demographics		
Age		
Mean—year	47.6 ± 20.1	
< 18 year	91 (7.7)	6.2–9.3
18–59 year	732 (61.6)	58.8–64.4
≥60 year	366 (30.8)	28.2–33.5
Female	627(52.7)	50.0–55.5
Male	562(47.3)	44.5–50.0
Organ system involvement		
Cardiovascular	996 (83.8)	81.3–85.8
Respiratory	659 (55.4)	52.5–58.3
Central nervous system	596 (50.1)	47.4–53.1
Mucocutaneous	563 (47.4)	44.6–50.5
Gastrointestinal tract	372 (31.3)	28.8–33.9
Severity of anaphylaxis		
Mild to moderate	160 (13.5)	11.5–15.6
Severe	1029 (86.5)	84.4–88.5
Outcome		
ICU admission	73 (6.1)	4.9–7.8
Death	39 (3.3)	2.3–4.3

*ED* emergency department, *ICU* intensive care unit

The majority of patients (83.8%) experienced cardiovascular anaphylactic symptoms; the percentage of patients who developed mucocutaneous compromise, respiratory compromise, central nervous symptoms were 47.4, 55.4, and 50.1%, respectively. Gastrointestinal anaphylactic symptoms occurred in 31.3% of the cases. Overall, 73 (6.1%) of the patients were admitted to intensive care units (ICU), and 39 (3.3%) patients died during their hospitalizations as a result of anaphylaxis.

### Drug triggers

A total of 249 individual drugs were involved in the anaphylactic cases analyzed, classified into 23 pharmacotherapeutic groups and 53 subgroups. A total of 1145 (96.3%) cases were attributed to single drugs, and 44 (3.7%) were attributed to “Associations” (Table 2). While various drug triggers were reported, the main four general categories for DIAs were antibiotics (39.3%), traditional Chinese medicines (TCM, 11.9%), radiocontrast agents (11.9%) and antineoplastic agents (10.3%).

**Table 2:** Pharmacotherapeutic groups and subgroups involved of drug-induced anaphylaxis

Drugs	Value, no. (%)	95% CI
Total	1189	
Antibiotics	467 (39.3)	36.7–42.1
β-lactams	275 (23.1)	21.0–25.5
Fluoroquinolones	138 (11.6)	9.8–13.4
Macrolides	28 (2.4)	1.6–3.3
Other antibiotics^a^	26 (2.2)	1.3–3.0
TCM	141 (11.9)	10.2–14.3
TCM (injection)	135 (11.4)	9.5–13.2
TCM (oral)	5 (0.4)	0.1–0.8
TCM (topical)	1 (0.1)	0.0–0.3
Radiocontrast agents	141 (11.9)	10.2–14.3
X-ray contrast media, iodinated	113 (9.5)	7.8–11.3
MRI contrast media	12 (1.0)	0.5–1.6
Ophthalmic medicines	10 (0.8)	0.3–1.4
Ultrasound contrast agents	4 (0.3)	0.1–0.8
Other^b^	2 (0.2)	0.0–0.4
Antineoplastics	122 (10.3)	8.8–12.4
Taxanes	73 (6.1)	4.9–7.6
Platinum compounds	40 (3.4)	2.4–4.5
Cytotoxic antibiotics	5 (0.4)	0.1–0.8
Alkylating agents	2 (0.2)	0.0–0.4
Teniposide	1 (0.1)	0.0–0.3
Asparaginase	1 (0.1)	0.0–0.3
Blood products, Biologics and plasma substitutes	74 (6.2)	4.9–7.7
Blood products and Biologics	37 (3.1)	2.2–4.2
Plasma substitutes	25 (2.1)	1.3–3.0
Monoclonal antibodies	12 (1.0)	0.5–1.6
Anesthetics	25 (2.1)	1.3–3.0
Local anesthetics	10 (0.8)	0.3–1.4
NMBAs	12 (1.0)	0.5–1.6
General anesthetics	3 (0.3)	0.0–0.6
Vaccines, immunoglobulins and antiserums	23 (1.9)	1.2–2.8
Immunoglobulins and antiserums	19 (1.6)	0.9–2.4
Vaccines	4 (0.3)	0.1–0.7
Nutrition and vitamins	22 (1.9)	1.1–2.7
Vitamins and minerals	16 (1.3)	0.8–2.1
Amino acids and fat emulsions	6 (0.5)	0.2–0.9
Immune-modulators	21 (1.8)	1.0–2.5
Immunostimulants	16 (1.3)	0.8–1.9
Immunosuppressants	5 (0.4)	0.1–0.8
Blood system medications	20 (1.7)	0.9–2.4
Antifibrinolytic agents and hemostatics	11 (0.9)	0.4–1.5
Iron (injection)	6 (0.5)	0.2–1.0
Anticoagulants	2 (0.2)	0.0–0.4
Antiplatelet agents	1 (0.1)	0.0–0.3
Analgesics, anti-inflammatory drugs and antipyretics	17 (1.4)	0.8–2.2
NSAIDs	11 (0.9)	0.4–1.6
Opioids	5 (0.4)	0.1–0.8
Paracetamol	1 (0.1)	0.0–0.3
Hormones, other endocrine medicines	16 (1.3)	0.8–2.1
Corticosteroids	11 (0.9)	0.4–1.5
Hypothalamic and pituitary hormones	4 (0.3)	0.1–0.7
Insulins	1 (0.1)	0.0–0.3
Prostaglandins	10 (0.8)	0.3–1.4
Cardiovascular medications	11 (0.9)	0.4–1.5
Vasodilators	5 (0.4)	0.1–0.8
Antiarrhythmics	3 (0.3)	0.0–0.6
ACEI/BB	2 (0.2)	0.0–0.4
Statins	1 (0.1)	0.0–0.3
Gastrointestinal medicines	8 (0.7)	0.3–1.1
H2 receptor blockers	3 (0.3)	0.0–0.6
Laxatives	2 (0.2)	0.0–0.4
PPI	1 (0.1)	0.0–0.3
Antacids	1 (0.1)	0.0–0.3
Antiemetics	1 (0.1)	0.0–0.3
Antidotes	7 (0.6)	0.3–1.1
Antivirals	5 (0.4)	0.1–0.8
Antifungals	3 (0.3)	0.0–0.6
Psychotherapeutic medicines	3 (0.3)	0.0–0.6
Antidementia medicines	2 (0.2)	0.0–0.4
Antipsychotic medications	1 (0.1)	0.0–0.3
Respiratory medications	3 (0.3)	0.0–0.6
Expectorants	2 (0.2)	0.0–0.4
Bronchodilators	1 (0.1)	0.0–0.3
Calcium regulating drugs	1 (0.1)	0.0–0.3
Associations^c^	44 (3.7)	2.7–4.9
Others^d^	5 (0.4)	0.1–0.8

*TCM* traditional Chinese medicine, *NSAIDs* non-steroidal anti-inflammatory drugs, *MRI* magnetic resonance imaging, *NMBAs* neuromuscular blocking agents, *ACEI* angiotensin converting enzyme inhibitor; *BB* beta-blocker, *PPI* proton pump inhibitor

^a^Other antibiotics included aminoglycosides, clindamycin, vancomycin, and metronidazole

^b^Other radiocontrast agent was indocyanine green injection

^c^Associations were defined as those cases in which more than one medication was suspected to cause the anaphylaxis. Details of anaphylaxis induced by associations were listed in Appendix 3

^d^“Others” category included monosialotetrahexosylganglioside sodium for injection, sodium deoxyribonucleotide injection, cerebroprotein hydrolysate for injection, and coenzyme A for injection

Antibiotics held the leading trigger medications of drug-induced anaphylaxis (467/1189, 39.3%). Among the antibiotics, the top three sub-groups included beta-lactams (275/467, 58.9%), fluoroquinolones (138/467, 29.6%), and macrolides (28/467, 6.0%) (Table 3). Within the beta lactams in particular, cephalosporins (161/275, 58.5%) were identified the most followed by beta-lactam/beta-lactamase inhibitors (72/275, 26.2%) and penicillins (37/275, 13.5%) (Table 3).

**Table 3:** Drugs of antibiotic-induced anaphylaxis

Antibiotics	ATC-codes	All Patients (n = 467) Value, no. (%)
β-Lactam antibiotics		275 (58.9)
Cephalosporins		161 (34.5)
First-generation	J01DB	13 (2.8)
cefradine	J01DB09	4 (0.9)
cefalexin	J01DB01	4 (0.9)
cefazolin	J01DB04	3 (0.6)
cefadroxil	J01DB05	1 (0.2)
cefathiamidine	NA	1 (0.2)
Second-generation	J01DC	86 (18.4)
cefuroxime	J01DC02	54 (11.6)
cefmetazole	J01DC09	18 (3.9)
cefoxitin	J01DC01	6 (1.3)
cefotiam	J01DC07	3 (0.6)
cefminox	J01DC12	2 (0.4)
cefamandole	J01DC03	2 (0.4)
cefaclor	J01DC04	1 (0.2)
Third-generation	J01DD	58 (12.4)
ceftriaxone	J01DD04	35 (7.5)
ceftazidime	J01DD02	10 (2.1)
ceftizoxime	J01DD07	6 (1.3)
cefoperazone	J01DD12	4 (0.9)
cefotaxime	J01DD01	2 (0.4)
cefdinir	J01DD15	1 (0.2)
Fourth-generation	J01DE	4 (0.9)
cefepime	J01DE01	4 (0.9)
β-lactam + β-lactamase inhibitors		72 (15.4)
cefoperazone + sulbactam	J01DD62	45 (9.6)
piperacillin + sulbactam	J01CR05	12 (2.6)
piperacillin + tazobactam	J01CR05	5 (1.1)
ampicillin + sulbactam	J01CA51	5 (1.1)
amoxicillin + clavulanic acid	J01CR02	3 (0.6)
imipenem + cilastatin	J01DH51	1 (0.2)
amoxicillin + sulbactam	J01CR02	1 (0.2)
Penicillins	J01C	37 (7.9)
benzylpenicillin	J01CE01	23 (4.9)
Penicillins with extended spectrum (aminopenicillins)	J01CA	14 (3.0)
azlocillin	J01CA09	5 (1.1)
amoxicillin	J01CA04	5 (1.1)
mezlocillin	J01CA10	4 (0.9)
Others		5 (1.1)
aztreonam	J01DF01	3 (0.6)
latamoxef	J01DD06	2 (0.4)
Fluoroquinolones	J01MA	138 (29.6)
levofloxacin	J01MA12	82 (17.6)
moxifloxacin	J01MA14	23 (4.9)
gatifloxacin	J01MA16	14 (3.0)
pefloxacin	J01MA03	5 (1.1)
ofloxacin	J01MA01	4 (0.9)
fleroxacin	J01MA08	4 (0.9)
ciprofloxacin	J01MA02	4 (0.9)
lomefloxacin	J01MA07	2 (0.4)
Macrolides	J01FA	28 (6.0)
azithromycin	J01FA10	28 (6.0)
clindamycin	J01FF01	14 (3.0)
Aminoglycosides	J01G	8 (1.7)
etimicin	NA	7 (1.5)
gentamicin	J01GB03	1 (0.2)
vancomycin	J01XA01	3 (0.6)
metronidazole	J01XD01	1 (0.2)

*ATC* anatomical therapeutic chemical, *NA* not available

There were 141 DIA cases (11.9%) induced by TCMs, most cases involving TCM injections (135/141, 95.7%), with the remaining cases including oral or topical TCM formulations. A total of 36 different TCM injections were identified. These injections were mainly used for the treatment of cardiovascular and cerebrovascular disease, digestive system disease, respiratory system disease, and cancer. *Ciwujia* was the leading cause followed by *Qingkailing*, *Houttuynia cordata*, *Shuxuening*, *Shuanghuanglian*, *Chuanhuning*, *Safflower* and *Yinxingdamo* (Tables 2, 4). Other TCM injections (53 cases) were listed in Appendix 1 in the electronic supplementary materials.

**Table 4:** Drugs of radiocontrast-induced anaphylaxis, TCM injection-induced anaphylaxis, and antineoplastics-induced anaphylaxis

Drugs	ATC-codes	Value, no. (%)
Radiocontrast agents (n = 141)		
iopromide	V08AB05	52 (36.9)
iohexol	V08AB02	26 (18.4)
iopamidol	V08AB04	16 (11.3)
fluorescein sodium	NA	10 (7.1)
gadopentetic acid (gadopentetate dimeglumine)	V08CA01	9 (6.4)
ioversol	V08AB07	7 (5.0)
iobitridol	V08AB11	5 (3.5)
iodixanol	V08AB09	5 (3.5)
sulfur hexafluoride	V08DA05	4 (2.8)
diatrizoic acid (meglumine diatrizoate)	V08AA01	2 (1.4)
indocyanine green	NA	2 (1.4)
gadobenic acid (gadobenate dimeglumine)	V08CA08	1 (0.7)
gadodiamide	V08CA03	1 (0.7)
gadoteric acid (gadoterate meglumine)	V08CA02	1 (0.7)
TCM injections (n = 135)		
Ciwujia	NA	21 (15.6)
Qingkailing	NA	16 (11.9)
Houttuynia cordata	NA	12 (8.9)
Shuxuening	NA	11 (8.1)
Shuanghuanglian	NA	6 (4.4)
Chuanhuning	NA	6 (4.4)
Safflower	NA	5 (3.7)
Yinxingdamo	NA	5 (3.7)
Others^a^	NA	53 (39.3)
Antineoplastics (n = 122)		
paclitaxel	L01CD01	68 (55.7)
oxaliplatin	L01XA03	18 (14.8)
carboplatin	L01XA02	13 (10.7)
cisplatin	L01XA01	7 (5.7)
docetaxel	L01CD02	5 (4.1)
cyclophosphamide	L01AA01	2 (1.6)
doxorubicin	L01DB01	2 (1.6)
nedaplatin	NA	2 (1.6)
topotecan	L01XX17	1 (0.8)
epirubicin	L01DB03	1 (0.8)
bleomycin A5	NA	1 (0.8)
mitomycin	L01DC03	1 (0.8)
asparaginase	L01XX02	1 (0.8)

*TCM* traditional Chinese medicine, *ATC* anatomical therapeutic chemical, *NA* not available

^a^Only the top 8 common TCMs were listed; detailed information on the Others category was presented in Appendix 1 in the electronic supplementary materials

Radiocontrast agents were reported 141 times, and the top three were contrast media used in X-ray (113/141, 80.1%), magnetic resonance imaging (12/141, 8.5%) and ophthalmic procedures (10/141, 7.1%) (Tables 2, 4).

Among the DIA cases caused by antineoplastic drugs (122/1189, 10.3%), paclitaxel (68/122, 55.7%) and platinum-based antineoplastics (40/122, 32.8%) were important contributors (Tables 2, 4). The remaining identified DIA cases (274) were listed in Table 2.

There were 149 anaphylaxis cases occurred during perioperative procedures, and the top three drug groups involved were antibiotics (43.0%), radiocontrast agents (14.8%) and plasma substitutes (9.4%) (Appendix 2).

Most causative drugs were administered by the intravenous route (86.4%), oral route (5.4%), intramuscular route (3.0%), subcutaneous route (1.6%), and intra-arterial route (1.4%). There were 12 cases of anaphylactic reactions occurred during intradermal tests.

Of the 44 DIA cases caused by the association of two or more drugs, 14 (31.8%) anaphylaxis cases involved one or more TCM combined with one or more other non-TCM, 12 (27.3%) occurred during general anesthesia, and 21 (47.7%) included antibiotics (Appendix 3).

### Severity and anaphylaxis-related deaths

Among the 1189 DIA cases, 1029 (86.5%) were considered as severe (grade 3) and 39 (3.3%) were fatal. Mortality was due mainly to the following top four medication groups: antibiotics (13/39, 33.3%), radiocontrast agents (12/39, 30.8%), antineoplastic agents (4/39, 10.3%), TCM injections (3/39, 7.7%). The detailed information was listed in Table 5.

**Table 5:** Description of the anaphylaxis-related deaths

Variable	ATC-codes	All patients (n = 39) value, no. (%)
Age		
Mean—year		53.8 ± 19.1
< 18 year		1 (2.6)
18–59 year		20 (51.3)
≥60 year		18 (46.1)
Female		14 (35.9)
Antibiotics		13 (33.3)
cefuroxime	J01DC02	5 (12.8)
levofloxacin	J01MA12	3 (7.7)
ceftriaxone	J01DD04	1 (2.6)
ceftizoxime	J01DD07	1 (2.6)
cefepime	J01DE01	1 (2.6)
piperacillin + sulbactam	J01CR05	1 (2.6)
clindamycin	J01FF01	1 (2.6)
Radiocontrast agents		12 (30.8)
ioversol	V08AB07	4 (10.3)
iohexol	V08AB02	3 (7.7)
iopromide	V08AB05	2 (5.1)
iopamidol	V08AB04	1 (2.6)
gadopentetic acid (gadopentetate dimeglumine)	V08CA01	1 (2.6)
fluorescein sodium	NA	1 (2.6)
Antineoplastics		4 (10.3)
paclitaxel	L01CD01	2 (5.1)
oxaliplatin	L01XA03	1 (2.6)
asparaginase	L01XX02	1 (2.6)
TCM injections		3 (7.7)
Houttuynia cordata	NA	2 (5.1)
Chuanhuning	NA	1 (2.6)
plasma substitutes		2 (5.1)
hydroxyethyl starch	B05AA07	1 (2.6)
dextran	B05AA05	1 (2.6)
Vaccine	J07BB03	1 (2.6)
protamine	V03AB14	1 (2.6)
articaine	N01BB08	1 (2.6)
doxofylline	R03DA11	1 (2.6)
Associations^a^		1 (2.6)

*TCM* traditional Chinese medicine, *ATC* anatomical therapeutic chemical, NA not available

^a^Associations were Moxifloxacin hydrochloride and sodium chloride injection, and Ambroxol hydrochloride for injection

## Discussion

To our knowledge, this is the first analysis of drug-induced anaphylaxis in the hospital setting in China. Using the Beijing Pharmacovigilance Database, our study shows that there were 1189 DIA cases in clinical settings over a decade in Beijing, China, accounting for 12.6% of all ADE reports collected by the BPD. The percentage of DIA in Beijing, China is higher than the percentage of DIA in Portugal (5–7%) spanning over a decade [8]. Patients aged between 18 and 59 years (61.6%) had the highest frequency of DIA among all three age groups, which is consistent with a recent study in China by Jiang et al. [15]. Similar to most studies [8, 9, 16], females had a higher frequency of anaphylaxis compared to males in our analysis. A high proportion of patients developed cardiovascular symptoms (83.8%), and the mortality rate of 3.3% is comparable to previous studies [17, 18].

### Antibiotic-induced anaphylaxis

Of all DIAs reported in BPD and included within our analysis, antibiotics attributed to the most common cause of DIA (39.3%), comparable to the incidence of antibiotic-induced anaphylaxis within the U.S. (40.5%) [6]. However, antibiotic-induced anaphylaxis occurred more frequently than reports from Korea (10.5%) [7] and Portugal (16.7%) [8]. In agreement with previous reports, anaphylaxis from β-lactam antibiotics (58.9%) were reported more frequently than non-β-lactam antibiotics [19]. Cephalosporins accounted for the majority of β-lactam antibiotic-related anaphylaxis followed by beta-lactam/beta-lactamase inhibitors and penicillins. This may be explained partly by the greater use of cephalosporins in Chinese hospitals [20]. The likelihood of anaphylaxis from penicillins can be assessed by skin tests [19]. However, routine intradermal skin testing of a cephalosporin may not be useful for predicting an immediate hypersensitivity because of the extremely low test sensitivity, which was confirmed by a recent retrospective study conducted by Yoon et al. [21]. Fluoroquinolone antibiotics also accounted for a high proportion of all 1189 anaphylaxis cases (11.6%), which is consistent with the frequency (11.7%) found by Faria et al. [16]. The high frequency of anaphylaxis from fluoroquinolones maybe due to the overuse of fluoroquinolone antibiotics in China [20], and the hypersensitivity to quinolones is less likely to be determined from skin testing [2].

Given that antibiotics are the most common trigger for anaphylaxis, the importance of inquiring and documenting patient’s medication allergic history cannot be underestimated. This is the area pharmacists could contribute to patient safety. Clinical symptoms and signs related to anaphylaxis should be closely monitored when antibiotics, especially cephalosporins and fluoroquinolones, are administered to patients in the hospital setting.

### TCM-induced anaphylaxis

The use of TCM was the second most common drug trigger in our analysis, and almost all (95.7%) TCM-related anaphylactic cases were from injectable TCM formulations. The result is similar to a study reported by Jiang et al. [15]. TCM is unique to the Chinese population and therefore the DIAs associated with TCM is unique to that population as a result. Along with extensive indications for TCM, there is greater use of TCM injections compared to other countries. In our review, 36 different TCM injections were identified resulting in 11.4% of the anaphylaxis cases, among which three cases were fatal. The four most common triggers were injections of *Ciwujia, Qingkailing, Houttuynia cordata* and *Shuxuening*. The results were similar to those of Jiang et al., who found the most common triggers were injections of *Qingkailing, Shuanghuanglian* and *Houttuynia cordata* [15].

TCM injection is extracted from Chinese herbs, which may contain one or several active ingredients. The high frequency of anaphylactic cases related to TCM injections may be explained as follows: (1) the components of a TCM injection are relatively complex and most formulations have not been thoroughly analyzed and identified; (2) a TCM injection also contains various additives, such as pigment, tannin, starch and protein, and these additives may trigger the body’s immune system and cause anaphylaxis [22]; (3) quality control of TCM injections is relatively difficult due to the formulation complexity, and impurities may cause anaphylaxis as well; and (4) drug interactions between TCM injections and other medications should also be considered. Of the 135 patients who suffered anaphylaxis induced by TCM injections, patients’ age ranged from 4 to 90 years: children under 18 years (5%), adults between 18 and 59 years (58.6%), and adults over 60 years (36.4%). This is consistent with a previous study reporting that patient of any age can suffer anaphylaxis induced by TCM injections [22]. Therefore, caution should be exercised before considering the use of TCM and all patients regardless of age should be closely monitored during TCM administration.

### Radiocontrast-induced anaphylaxis

Our study found that radiocontrast agents were the third most common cause (11.9%) of DIA, coinciding with the frequency of previous studies in Korea (12.0%) [7]. However DIA through radiocontrast agents was reported less frequently than that in the United States (30.4%) [6]. Of these radiocontrast-induced anaphylaxis cases, the majority (80.1%) were caused by iodine-based contrast agents. The number of anaphylaxis cases induced by iopromide (36.9%) were greater than any other non-ionic iodinated contrast agents, which is similar to the results from a previous study [23]. A recent retrospective study from Korea indicated that among the anaphylactic patients, iopromide was associated with more severe anaphylaxis with hypotension [24].

Patients with allergies, asthma, renal insufficiency, anxiety, significant cardiac disease and other miscellaneous risk factors may be at an increased risk for anaphylactoid contrast reactions [25]. Patient’s medical history should be collected and the risk of contrast media induced anaphylactic reactions should be assessment before contrast media administration. More importantly, appropriate resuscitative equipment should be available to treat anaphylactic reactions promptly. Premedications such as corticosteroids should also be used for pretreatment of “at-risk” patients who require a contrast-enhanced examination [25].

### Antineoplastics-induced anaphylaxis

Antineoplastic agents were another frequent cause of DIA in the current study, consistent with those reported in literature [6, 8]. This may be related to the increasing chemotherapy use with ever increasing cancer prevalence in Beijing, China [26]. Paclitaxel accounted for 55.7% of antineoplastics-induced anaphylaxis, and the high frequency may be related to the solubilizer, polyoxyethylene castor oil, which can induce anaphylaxis [27]. We also found that platinum-based agents accounted for 32.8% of all antineoplastics-induced anaphylaxis cases. Among these agents, oxaliplatin was a major trigger accounting for 1.5% of all 1189 DIA cases, and this finding is consistent with a published study [28].

Although a recent study from Nonna et al. found that for patients with carboplatin induced hypersensitivity reaction, the use of oxaliplatin maybe a safer alternative [29], patients should be carefully monitored for signs and symptoms of anaphylaxis with any platinum-based chemotherapeutic agent.

### Limitations

Our retrospective analysis was based on self-reported cases by health care professionals from the BPD, and therefore our study has the following limitations: (1) lack of the frequency of causative drug use as we could not obtain either prescription or reimbursement data; (2) we could not assess the prevalence of DIA in the region studied as we do not have the information of the total patient base; (3) potential reporting bias may exist: majority of reported cases were hospitalized patients in the non-ED setting, and only severe anaphylactic cases may have been reported; (4) we may not have included all DIA cases in the BPD: cases missed if clinicians did not report using the terms related to allergy or anaphylaxis or hypersensitivity (e.g. a patient with wheeze, vomiting, bronchospasm but was not described as “allergy” by clinicians when reporting to the BPD). In addition, some reported cases were not included due to insufficient information. Despite these limitations, the method we have taken should be robust against a range of potential biases: rigorous inclusion/exclusion criteria were utilized and all potential anaphylaxis cases were adjudicated by trained physician/allergists; and only patients with confirmed anaphylaxis and complete data record were included in the analysis.

## Conclusion

This first detailed analysis of DIA case reports from 2004 to 2014 in Chinese patients provides valuable information to clinicians. Antibiotics, TCM, radiocontrast media and antineoplastic agents are the most common causes of DIA cases. The majority of DIA cases are considered to be severe with a high mortality rate of 3.3%. Pharmacists should be working closely with prescribers to assess each patient’s risks of developing anaphylaxis when drug therapy is involved, and to provide prompt treatment and resuscitations to reduce the morbidity and mortality when anaphylaxis occurs.

## Electronic supplementary material

Below is the link to the electronic supplementary material.

Supplementary material 1 (DOCX 21 kb)
