# Supplementary material for: Drug-induced anaphylaxis in China: a 10 year retrospective analysis of the Beijing Pharmacovigilance Database
Source: Int J Clin Pharm. 2017 Oct 31;40(5):1349–58. doi: 10.1007/s11096-017-0535-2 (PMC6208584; doi:10.1007/s11096-017-0535-2)
Supplement: Supplementary file 1 — Supplementary material 1 (DOCX 21 kb) [file 11096_2017_535_MOESM1_ESM.docx]

**International Journal of Clinical Pharmacy**

**Supplementary Appendix**

**Supplement to:**

**Drug-induced Anaphylaxis in China:** A 10 year Retrospective Analysis of the Beijing Pharmacovigilance Database

Contents

**Appendix 1 – Details of other TCM injections**

**Appendix 2 – Description of drug-induced anaphylaxis occurring during perioperative period**

**Appendix 3 – Details of anaphylaxis induced by associations**

**Appendix 1 – Details of other TCM injections**

| **TCMs^a^** | **All Patients**  **(n=135)**  **Value, no. (%)** |
| --- | --- |
| Compound glycyrrhizin injection | 4 (3.0) |
| Tanreqing injection | 4 (3.0) |
| Shuxuetong injection | 3 (2.2) |
| Xingnaojing injection | 3 (2.2) |
| Xueshuantong injection | 3 (2.2) |
| Luguaduotai injection | 3 (2.2) |
| Xuesaitong injection | 3 (2.2) |
| Sulfotanshinone sodium injection | 3 (2.2) |
| Elemene injection | 2 (1.5) |
| Ligustrazine hydrochloride for injection | 2 (1.5) |
| Danhong injection | 2 (1.5) |
| Compound kushen injection | 2 (1.5) |
| Alginic sodium diester and sodium chloride injection | 2 (1.5) |
| [Salvia miltiorrhiza](http://www.baidu.com/link?url=eVPqTiHqI9wuAl4iNuHJf0zEmoAktbwvoxiYFEmvao6PaP_DCg59-Kc3fL4bi_qeySsUbBWyy0Q6a9JBvKdZea) injection | 2 (1.5) |
| Reduning injection | 2 (1.5) |
| Xiaoaiping injection | 1 (0.7) |
| Compound ammonium glycyrrhizinate injection | 1 (0.7) |
| Magnesium isoglycyrrhizinate injection | 1 (0.7) |
| Mailuoning injection | 1 (0.7) |
| Kuhuang injection | 1 (0.7) |
| Cervus and cucumis polypeptide for injection | 1 (0.7) |
| Shenmai injection | 1 (0.7) |
| Sodium aescinate for injection | 1 (0.7) |
| Kudiezi injection | 1 (0.7) |
| Bupleurum injection | 1 (0.7) |
| Xiyanping injection | 1 (0.7) |
| Kanglaite injection | 1 (0.7) |
| Herba Erigerontis injection | 1 (0.7) |

^a^ Detailed information on the Others category was listed; the top 8 common TCMs were listed Table 4.

TCM Traditional Chinese Medicine

**Appendix 2 – Description of drug-induced anaphylaxis occurring during perioperative period**

| **Drugs** | **All Patients**  **(n=149)**  **Value, no. (%)** |
| --- | --- |
| Antibiotics | 64 (43.0) |
| Radiocontrast agents | 22 (14.8) |
| Plasma substitutes | 14 (9.4) |
| Associations^a^ | 12 (8.1) |
| hemostatics | 5 (3.4) |
| Blood products and Biologics | 4 (2.7) |
| NSAIDs | 4 (2.7) |
| Prostaglandins | 4 (2.7) |
| NMBAs | 3 (2.0) |
| General anesthetics | 3 (2.0) |
| Local anesthetics | 2 (1.3) |
| Opioids | 2 (1.3) |
| Corticosteroids | 2 (1.3) |
| Immunostimulants | 2 (1.3) |
| Immunoglobulin | 1 (0.7) |
| Low molecular weight heparin | 1 (0.7) |
| Vasodilators | 1 (0.7) |
| Hypothalamic and pituitary hormones | 1 (0.7) |
| Fat emulsion | 1 (0.7) |
| Cerebroprotein hydrolysate | 1 (0.7) |

^a^ Associations were defined as those cases in which more than one medication was suspected to cause the anaphylaxis.

NMBAs neuromuscular blocking agents, NSAIDs non-steroidal anti-inflammatory drugs

**Appendix 3 - Details of anaphylaxis induced by associations^a^**

| **Number** | **Drugs** |
| --- | --- |
| 1 | Propofol injection  Midazolam injection  Cisatracurium besilate for injection |
| 2 | Propofol injection  Atracurium besilate for injection |
| 3 | Rocuronium injection  Propofol injection |
| 4 | Ciprofloxacin lactate injection  Ornidazole injection |
| 5 | Atropine sulfate injection  Rocuronium injection  Ketamine hydrochloride injection  Remifentanil hydrochloride for injection  Propofol injection |
| 6 | Sevoflurane for inhalation  Rocuronium injection |
| 7 | Lidocaine hydrochloride injection  Rocuronium injection  Propofol medium and long chain fat emulsion injection  Sufentanil citrate injection |
| 8 | Etomidate fat emulsion injection  Cisatracurium besylate for injection |
| 9 | Cefuroxime sodium for injection  Metronidazole disodium phosphate for injection |
| 10 | Midazolam injection  Tropisetron hydrochloride injection  Propofol injection  Cefuroxime sodium for injection  Sufentanil citrate injection |
| 11 | Hydroxycthyl starch injection  Methylprednisolone sodium succinate for injection  Vecuronium bromide for injection |
| 12 | Ranitidine bismuth citrate capsules  Levofloxacin hydrochloride tablets  Domperidone tablets |
| 13 | Compound glycyrrhizin injection  Coenzyme complex for injection  Reduced glutathione for injection  Polyene phosphatidylcholine injection |
| 14 | Propofol injection  Fentanyl citrate injection  Midazolam injection  Remifentanil hydrochloride for injection  Flurbiprofen axetil injection  Succinylated gelatin injection  Atracurium besilate injection |
| 15 | Ornidazole for injection  Ciprofloxacin lactate and sodium chloride injection  Lidocaine hydrochloride injection |
| 16 | Lysine acetylsalicylate for injection  Ceftriaxone sodium for injection |
| 17 | Vitamin C yinqiao tablets  Levofloxacin mesylate tablets  Reyanning granules |
| 18 | Longxuejie capsules  Ibuprofen and codeine phosphate tablets  Diosmin tablets |
| 19 | Calcium gluconate injection  Vitamin C injection  Potassium magnesium aspartate for injection  Vitamin complex tablets |
| 20 | Ceftriaxone sodium for injection  Ambroxol hydrochloride injection  Vitamin C injection |
| 21 | Amoxicillin capsules  Metamizole sodium tablets  Aspirin effervescent tablets  Compound paracetamol and amantadine hydrochloride tablets |
| 22 | Azithromycin lactobionate for injection  Tanreqing injection |
| 23 | Cefotaxime sodium for injection  Aminophyiline injection |
| 24 | Qingkailing injection  Compound amidopyrine injection |
| 25 | Terfenadine tablets  Prednisone acetate tablets |
| 26 | Vitamin K_1_ injection  Kuhuang injection |
| 27 | Mailuoning injection  Lidocaine hydrochloride injection  Sodium bicarbonate injection |
| 28 | Adenosine disodium triphosphate injection  Coenzyme A and insulin for injection  Vitamin C for injection  Calcium dibutyryladenosine cyclophosphate for injection |
| 29 | Ligustrazine injection  Dextran 20 glucose injection |
| 30 | Shuxuening injection  Alprostadil injection |
| 31 | Compound diclofenac sodium and chlorphenamine maleate tablets  Sulfamethoxazole tablets  Sulfadiazine and trimethoprim tablets |
| 32 | Alprostadil injection  Yinxingdamo injection |
| 33 | Aescuven forte tablets  Moxifloxacin hydrochloride tablets |
| 34 | Yanhuning injection tablets  Cefoperazone for injection  Shuanghuanglian injection |
| 35 | Cefotaxime for injection  Creatine phosphate sodium for injection |
| 36 | Clindamycin phosphate injection  Aspisol for injection |
| 37 | Levothyroxine sodium tablets  Bisoprolol fumarate tablets |
| 38 | Benzylpenicillin sodium for injection  Qingkailing injection |
| 39 | Azithromycin for injection  Vitamin B_6_ injection |
| 40 | Hemocoagulase injection  Carbazochrome sodium sulfonate for injection |
| 41 | Ambroxol hydrochloride injection  Ceftazidime for injection |
| 42 | Moxifloxacin hydrochloride and sodium chloride injection  Ambroxol hydrochloride for injection |
| 43 | Ceftriaxone sodium for injection  Qingkailing injection |
| 44 | Unspecified^b^  Piperacillin sodium and sulbactam sodium for injection |

^a^ Associations were defined as those cases in which more than one medication was suspected to cause the anaphylaxis.

^b^ Incomplete data.
